# Toward a Kinh Vietnamese Reference Genome: Constructing a De Novo Genome Assembly Using Long-Read Sequencing and Optical Mapping

**DOI:** 10.3390/genes16050536

**Published:** 2025-04-29

**Authors:** Le Thi Dung, Le Tung Lam, Nguyen Hong Trang, Nguyen Vu Hung Anh, Nguyen Ngoc Nam, Doan Thi Nhung, Tran Huyen Linh, Le Ngoc Giang, Hoang Ha, Nguyen Quang Huy, Truong Nam Hai

**Affiliations:** 1 Institute of Biology, Vietnam Academy of Science and Technology (VAST), Hanoi 10072, Vietnam; dungcnshk55a@gmail.com (L.T.D.); letunglam1991@gmail.com (L.T.L.); trang.nt41@gmail.com (N.H.T.); nvuhunganh@gmail.com (N.V.H.A.); ngocgiangnam@gmail.com (N.N.N.); nhungdoan1905@gmail.com (D.T.N.); linh536.95@gmail.com (T.H.L.); hoanghapcb@ibt.ac.vn (H.H.); 2 Department of Life Sciences, University of Science and Technology of Hanoi (USTH), Vietnam Academy of Science and Technology (VAST), Hanoi 10072, Vietnam; nguyen-quang.huy@usth.edu.vn; 3 Terry Fox Laboratory, BC Cancer Research Centre (BCCRC), Vancouver, BC V5Z 1L3, Canada; 4 Comparative Genetics and Refinement, Biomedical Primate Research Centre (BPRC), 2288 GJ Rijswijk, The Netherlands; le@bprc.nl; 5 Department of Biotechnology, Graduate University of Science and Technology (GUST), Vietnam Academy of Science and Technology (VAST), Hanoi 10072, Vietnam; 6 Department of Omic Technologies and Application, Institute of Biology, Vietnam Academy of Science and Technology, Hanoi 10072, Vietnam

**Keywords:** Kinh Vietnamese genome, de novo assembly, long-read sequencing, optical mapping, Kinh structural variants

## Abstract

**Background:** Population-specific reference genomes are essential for improving the accuracy and reliability of genomic analyses across diverse human populations. Although Vietnam ranks as the 16th most populous country in the world, with more than 86% of its population identifying as Kinh, studies specifically focusing on the Kinh Vietnamese reference genome remain scarce. Therefore, constructing a Kinh Vietnamese reference genome is valuable in the genetic research of Vietnamese. **Methods:** In this study, we combined PacBio long-read sequencing and Bionano optical mapping data to generate a de novo assembly of a Kinh Vietnamese genome (VHG), which was subsequently polished using multiple Kinh Vietnamese short-read whole-genome sequences (WGSs). **Results:** The final assembly, named VHG1.2, comprised 3.22 gigabase pairs of high-quality sequence data, demonstrating high accuracy (QV: 48), completeness (BUSCO: 92%), and continuity (295 super scaffolds, super scaffold N50: 50 Kbp). Using multiple bioinformatic tools for variant calling, we observed significant variants when the population-specific reference VHG1.2 was used compared to the standard reference genome hg38. **Conclusions:** Overall, our genome assembly demonstrates the advantages of a long-read hybrid sequencing approach for de novo assembly and highlights the benefit of using population-specific reference genomes in population genomic analysis.

## 1. Introduction

Over the past few decades, human genomics and precision medicine have undergone tremendous advancements with the proliferation of multiple next-generation sequencing technologies. Short-read sequencing technologies in particular have been instrumental; they are routinely used in large-scale population analyses and molecular diagnostic applications owing to their high throughput, low cost, and high-accuracy output [1,2,3]. To this day, Illumina remains the most dominant sequencing platform on the market, responsible for generating more than 90% of the world’s sequencing data and serving as the primary sequencing platform for diagnostic genome sequencing globally [4,5,6]. However, these technologies have inherent limitations. Short-read sequencing requires alignment to a well-defined reference; if a short read is aligned to the wrong position, any difference between the reads and the reference can be misinterpreted as a mutation [3,7,8]. This is especially true regarding genomic variations between individuals or regions that short reads can only map to ambiguously [9,10,11].

Since the completion of the Human Genome Project and the release of the finished sequence in 2004, there remains a notable bias in genomic research, with a predominant focus on populations of European descent [12,13,14]. The most widely used human reference genomes, GRCh37 (hg19) and GRCh38 (hg38) are primarily constructed from individuals of European and African ancestry, resulting in the underrepresentation of other populations [15,16,17]. This imbalance introduces reference bias, where reads containing alternative alleles common in underrepresented populations are less likely to align correctly to the reference genome. This misalignment can lead to inaccurate variant calling and potentially misleading interpretation in downstream analyses [18,19,20].

To improve the reference accuracy for short-read sequencing analysis and to address the gap in representation, numerous groups across the world have constructed population-specific reference genomes from high-coverage, de novo assembled individual human genomes, such as the Chinese genomes [21,22,23], Korean [24,25], Danish [26], Swedish [27], and Japanese [8,28]. All of these genomes are continuously being upgraded to improve their completeness and specificity [29,30,31,32]. Subsequent analyses using these population-specific genomes revealed substantial differences in mapping and variant calling results [20,28,33]. These findings highlighted the necessity of developing population-specific reference genomes to enhance the reliability of genomic analyses across diverse human populations.

Long-read sequencing technologies have now achieved a level of accuracy and throughput that significantly expands their applications in genomics. Technologies such as single-molecule real-time (SMRT) sequencing by Pacific Biosciences and nanopore sequencing by Oxford Nanopore Technologies generate reads that can span tens of kilobases (Kbp) to megabases (Mbp) [1,34]. These long reads enable the resolution of complex and repetitive regions, facilitating more accurate and contiguous genome assemblies [35,36,37].

In addition to long-read sequencing, optical mapping has been intensively used to provide genome scaffolding and detect large-scale structural variations. Optical maps have an average molecule length of 225 Kbp and can easily span genomic regions that are difficult for DNA sequencing while not having the limitations of linked-read sequencing technologies [37]. Recent studies have demonstrated the success of combining long-read sequencing with optical mapping to produce high-quality de novo [27,38,39]. These combined approaches are particularly effective in creating de novo assemblies comparable to hg38 in terms of completeness and contiguity.

Previous studies have shown that the population structure of the Kinh Vietnamese (KHV), a population of 100 million people, has a main component that is most prevalent in the Chinese population and significantly diverges from not only European and African populations but also South Asian neighboring populations [10,40]. Thus, there have been calls to construct a population-specific reference genome to address the inherent biases in using hg38 reference for the Vietnamese population. A previous attempt to construct a KHV reference genome was limited by using exclusively Illumina’s short-read sequencing technology [41], which is not a viable platform to use in the context of creating a low-error assembly without a reference guide [42]. In this study, we combined PacBio HiFi sequencing and Bionano optical mapping to generate a high-quality de novo assembly of a Kinh Vietnamese genome and polish this construct using other Vietnamese short-read whole-genome sequences (WGSs) to create a population-specific genome. Though still in need of further work, our genome assembly represents a significant step toward a new population-specific reference for genomic analysis using short-read sequencing technologies in Vietnam.

## 2. Materials and Methods

### 2.1. Ethics Declarations

This study and sample collection were approved by the Scientific Council of the Institute of Biotechnology, Vietnam Academy of Science and Technology, on 1 June 2021.

### 2.2. Donor Selection

Three adult male Vietnamese volunteers, aged 30–59 years, were recruited for the study. This age range was chosen based on previous studies indicating it is the most biologically stable period with minimal genetic and health-related variability, which could influence genetic results [43]. The donors self-reported being healthy, with no inherited conditions, and were of Kinh ancestry, with their families having lived in Vietnam for more than eight generations. The selected donor has a healthy son without any inherited conditions or medical history.

### 2.3. Genomic DNA Preparation

Genomic DNA was extracted from fresh blood using the Blood & Cell Culture DNA Midi Kit (QIAGEN, Hilden, Germany) according to the manufacturer’s protocol. The resulting DNA molecules were 80–100 Kbp in size. DNA quality was assessed through gel electrophoresis, a Qubit 3 Fluorometer (Invitrogen, Waltham, MA, USA), and a 4200 TapeStation System (Agilent, Santa Clara, CA, USA) (Figure S1).

### 2.4. Pacbio HiFi Library Preparation and Sequencing

PacBio HiFi library preparation and sequencing were performed following the manufacturer’s standard protocol with minor modifications. Genomic DNA was sheared into fragments of 10–15 Kbp using g-tubes (Covaris, Brighton, UK) and processed using the SMRTbell Express Template Prep Kit 2.0 (PacBio). Each library preparation step was followed by cleanup using AMPure PB beads (PacBio, Menlo Park, CA, USA) and quality control using the Bioanalyzer 2100 system (Agilent). The final DNA library was loaded onto SMRT Cells (PacBio, Menlo Park, CA, USA) and sequenced using the PacBio Sequel and Sequel II systems, with sample setup in the SMRT Link portal version 9.0.

### 2.5. Bionano Optical Map Generation

Genome optical mapping was performed using the Saphyr system (Bionano Genomics) following the manufacturer’s protocol. A total of 1.5 mL of deep-frozen whole blood was shipped on dry ice to Bionano Genomics (San Diego, CA, USA) and processed according to the Bionano SP Blood and Cell Culture DNA Isolation protocol for 100X Human Genome Sample Analysis. Genome assembly, scaffolding, and structural variant detection were performed using Bionano Solve software version 3.2.

### 2.6. Genome Assembly of SMRT Sequencing Reads

The raw data were initially processed and evaluated through a primary analysis on a host computer before being transferred to a secondary analysis server via a local network. The data were analyzed using SMRTLink Portal v9.0 with a minimum predicted accuracy of 0.99. HiFi contig assembly was generated from HiFi reads using the HiFiasm [44,45] version 0.16.1-r37.

### 2.7. Hybrid Scaffold Assembly

Scaffolds constructed from Bionano data were combined with the HiFiasm contigs through Bionano Access version 1.7 on the Bionano platform to create super scaffolds assembly with parameters “Resolve Conflicts” and “Trim Overlapping Sequence = On” selected [38]. Conflicts between the assembly and optical maps were automatically resolved in favor of the optical maps. Later, a second round of super scaffolding was performed between the output of the hybrid process and the consensus optical map from the DLE-1 restriction enzyme [25,27,46]. The resulting hybrid scaffold assembly was termed super scaffolds.

### 2.8. Assembly Correction and Improvement with Pacbio HiFi Reads

The super scaffolds underwent polishing and correction with HiFi reads using Inspector [47] version 1.0.1, where it was applied with the error correction module and datatype PacBio HiFi to generate a draft assembly. The corrected and polished assembly was named VHG1.1.

### 2.9. Variant Calling for VHG

A small variant calling for VHG was performed in parallel using GATK version 4.4.0.0, according to a previous publication [38]. First, HiFi reads were mapped to reference genome hg38 using Minimap2 [48] version 2.24 (r1155), and the resulting data were converted to FASTQ format via Samtools to be used as input data for variant calling in the next steps. After removing duplicates, the alignment data were used to identify single-nucleotide polymorphisms (SNPs) and short insertions–deletions (indels) with default parameters. Then, filtered small variants were subjected to variant calling.

Structure variants (SVs) from HiFi reads were detected using Pbsv version 2.9.0 from PacBio (https://github.com/PacificBiosciences/pbsv (accessed on 6 May 2024)). The HiFi reads were aligned to the hg38 reference genome using Pbmm2 version 1.16.0 with default parameters and processed to identify SVs [46]. SVpack was used to filter the called SVs in VCF format using a series of filtering operations (https://github.com/PacificBiosciences/svpack (accessed on 7 May 2024)). Initially, only SV calls with a “PASS” flag in the “FILTER” column were filtered to obtain confident SV calls, and duplicates (if any) were removed for the following stage of the analysis. Next, the SVs were filtered based on the SV types (insertions, duplications, and deletions) to obtain each call set. Finally, the SVs were filtered for length ≥ 50 bp [27,46].

### 2.10. Polishing Assembly Using Illumina Short-Read Whole-Genome Sequences

To refine VHG for the Kinh Vietnamese population, the VHG1.1 assembly underwent further polishing using Jellyfish-based Assembly Sequence Polisher for Error Reduction (JASPER) tool with optimized parameters (-k 37, -t 24, and -p 4) to handle the inherent complexity of genomic assembly. Given the critical importance of representing the genetic diversity of the Kinh population, seven male Kinh Vietnamese (KHV) genomes from Ho Chi Minh City, Vietnam (HG01842, HG01846, HG02064, HG02076, HG02122, HG02058, HG01872), were sourced from the 1000 Genomes Project (1KGP) database (http://www.internationalgenome.org (accessed on 14 October 2024)). These genomes, totaling approximately 415 Gbp of Illumina sequencing data, were used to refine and enhance the VHG1.1 assembly toward population-representative reference, resulting in the VHG1.2 assembly.

### 2.11. Ordering and Aligning of Super Scaffolds to Chromosomes

The assembly was positioned onto the chromosomes of the hg38 reference genome using RagTag [49] version 2.1.0 with scaffold command [1,37]. Each group of assigned super scaffolds was double-checked through pairwise genome alignments to hg38 through Lastz [50] version 1.04.15 with the following parameters: --gfextend –chain –gapped. The data were visualized using R packages karyoploteR and GenomicRanges version 4.4.1.

Assemblies were mapped with hg38 and T2T references using Minimap2 version 2.24 (r1155) [48]. D-GENIES dotplot was used to quickly compare and visualize the alignment between the VHG1.2 assembly and hg38 or T2T references [51].

### 2.12. Genome Assembly Evaluation

Genome assemblies were evaluated by Inspector [47] version 1.0.1 with the datatype HiFi option for evaluation assembly using HiFi reads and the hg38 reference genome. Assembly completeness was assessed with Busco [52] version 5.5.0 with the -l parameter and primates_odb10 dataset. Statistical results were exported and compared to identify the highest-quality assembly. The final assembly was also evaluated with the QUAST toolkit [53] version 5.0.2.

### 2.13. Reference Genome Set

The hg38 (accession GCA_000001405.15) reference genome used in this study was described in a previous study [54]. It includes primary hg38 sequences (autosomes, chromosomes X and Y), mitochondrial genome, and unlocalized scaffolds that belong to a chromosome without a definitive location and order.

### 2.14. SNP and Indel Calling of KHV Genome

Three low-coverage WGS KHV genomes (HG01852, HG02070, and HG02079) were sourced from the 1KGP to be utilized for variant calling analysis. Common mapped reads between VGH1.1, VHG1.2, and hg38 were identified and extracted according to the method by Takayama et al. in 2021 [38]. In detail, the Illumina reads of each genome were mapped to each reference genome using bwa-mem [55] version 0.7.17-r1188 and processed with Samtools version 1.3.1. Then, mapped reads are extracted from bam files (-F 4 option), and their read names are isolated. The read names are then sorted and filtered for uniqueness using sort and uniq. Finally, common read names are identified using the awk command, and the corresponding reads are filtered from the bam files with the -N option in Samtools.

The output files of the above step were used for small variant calling with bioinformatic tools, have been evaluated, tested, and benchmarked, and are widely used in clinical genetics. First, we employed GATK version 4.4.0.0, the “industry standard” for short SNPs and indels calling [38,56]. The process starts by removing duplicate reads with MarkDuplicatesSpark and generates raw variant calls using HaplotypeCaller. Variants are categorized into SNPs and indels using SelectVariants, and both types were filtered based on quality metrics like Quality-by-Depth (QD < 2.0), low quality scores (QUAL < 30.0), high strand bias (FS > 200.0), or positional bias in reads (ReadPosRankSum < −20.0) with VariantFiltration. Finally, the remaining high-confidence variants were counted with VCF tools.

To prove that VHG1.2 is the first version of the Kinh Vietnamese population-specific reference genome, the same output files from Samtools were used to recall SNPs and indels with DeepVariant via Docker, a deep learning-based variant caller, following these default parameters: --model_type = WGS for whole-genome sequencing, --ref for the reference genome (hg38.fa, VHG1.1.fa, or VHG1.2.fa), and --reads for the BAM file containing mapped reads [57]. The variant calling data were exported to output_vcf and --output_gvcf files. The variants were counted using Excel for Microsoft 365.

### 2.15. Structural Variant Calling of KHV Genome

Cue, a deep learning SV calling framework [58], was utilized to detect SVs larger than 5 Kbp in KHV genomes (HG02134, HG01840, HG01852) from the 1KGP. The FASTQ sequencing reads were mapped with the hg38 reference genome and VHG1.2 by bwa-mem [50] version 0.7.18. The resulting BAM files and index files were processed using Cue with model cue.v2.pt.

### 2.16. Comparison VHG1.2 with East Asian Assemblies

To evaluate the genetic similarity of VHG1.2 to other East Asian assemblies, genome-to-genome alignments were performed using Minimap2 against hg38 and three publicly available East Asian reference genomes: AK1 (Korean, GCA_001750385.2), HX1 (Chinese, GCA_001708065.2), and JG1 (Japanese, GCA_014905855.1).

### 2.17. Data Visualization and Summary Statistics

Statistical analysis and data visualization were performed using Excel, R version 4.4.1, and Python version 3.9.16.

## 3. Results and Discussion

### 3.1. De Novo Assembly of a Kinh Vietnamese Genome

The study commenced with HiFi sequencing and optical mapping of DNA extracted from the blood of an anonymous Kinh Vietnamese donor with a normal karyotype who passed a general health examination (Figure S2). In total, 20X whole-genome coverage was achieved from SMRT and 1600 Gbp long-read sequencing with a mean subread length of 10.29 Kbp and an N50 subread length of 12.19 Kbp. Quality control measures using SMRTLink Portal v9.0 revealed 5.28 million subreads with a mean read length of 11.73 Kbp and read quality of Q36 (Tables S1 and S2).

A de novo assembly was performed from PacBio CCS reads, using HiFiasm software version 0.16.1-r37, generating 2198 HiFi contigs (N50 = 8.78 Mbp), with the longest contig at 78.82 Mbp and a total size of 3.01 Gbp (Table 1). In addition, a VHG mitochondrial sequence was assembled using a similar approach (Figure S3).

At the same time, an optical map with a depth coverage of 129.57 X was also generated (Table S3), as previously described, with a label density of 15,77 per 100 Kbp [38]. After filtering, this optical map was de novo assembled to produce a genome map. The size of the optical genome map was 5.8 Gbp, containing 1042 optical scaffolds. The N50 of the scaffolds was approximately 39.14 Mbp, and the longest scaffold was 108 Mbp (Table S4).

To obtain whole-genome assemblies, the HiFi contigs were aligned to the optical mapping scaffolds using the Bionano Access platform, forming super scaffolds [11,38]. This process resolved many gaps in the HiFi reads (Figure S4). The hybridization of 2198 HiFi contigs and 1042 Bionano scaffolds produced 295 super scaffolds, with an N50 of 50.6 Mbp (Table S4). When compared to other assemblies using the same method, our Kinh Vietnamese assembly (VHG) showed significant improvements in both sequencing and assembly metrics (Table 1). Notably, VHG had fewer super scaffolds than the most up-to-date JG1 hybrid assembly (295 and 624, respectively), suggesting that VHG is a more optimized scaffold-level assembly.

### 3.2. SNPs and Indels Detection of VHG’s HiFi Reads

The study identified 3,905,585 SNPs and 2,095,662 indels in the VHG by comparing HiFi reads with hg38 (Table S5). These numbers can be compared with 4,823,475 SNPs and 974,100 indels (length ≤ 100 bp) detected in the KHV [41] and 3,518,309 SNPs and 378,750 indels detected in the HX1 genome [21]. The differences in the number of small variants can be attributed to variations in sequencing technologies, variant calling approaches, and population characteristics.

**Figure 1:**
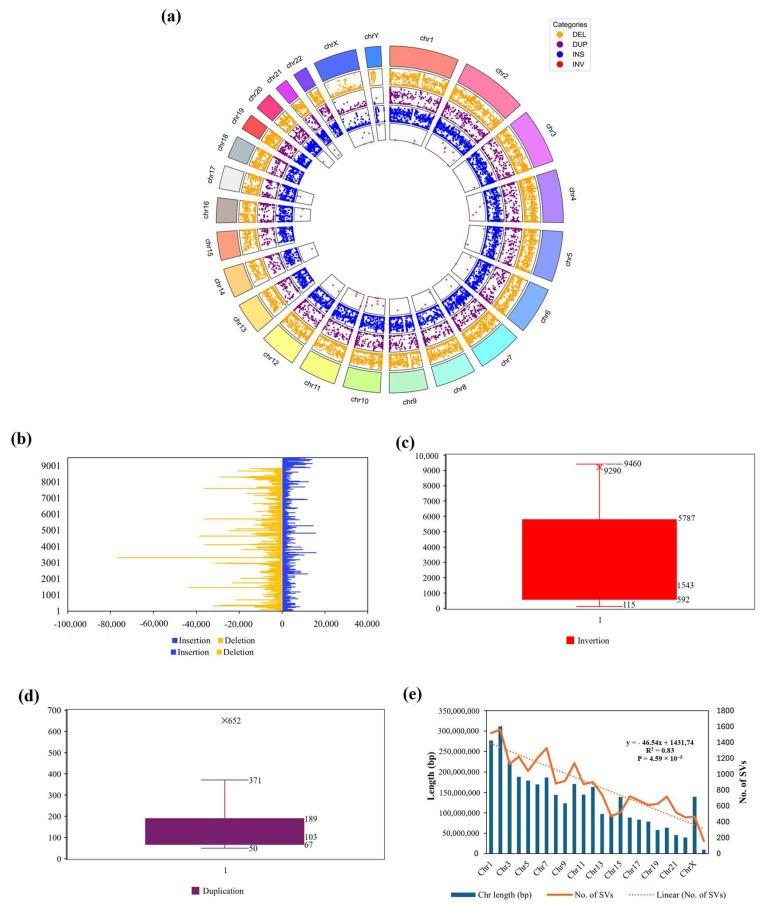
A summary of structural variants (SVs) detected in the VHG genome. (**a**) The distribution of insertions (blue), deletions (orange), inversions (red), and duplications (purple) between VHG and hg38 is displayed in a Circos plot. (**b**) The number and size of insertion and deletion SVs in VHG. (**c**,**d**) The number and size of inversions and duplications of SVs in VHG, respectively. The asterisk (*) represents the mean value of the data. The median value lies in the middle of the color box. (**e**) Correlation of chromosome length (dark teal column) with SV numbers (orange line). *p*-value refers to the significance of the statistical test (*p* < 0.05 corresponding significantly).

Structural variants (SVs) in the VHG were identified through a comparative analysis with the GRCh38 genome assembly. The analysis found a total of 21,760 SVs, with sizes starting at 50 bp, comprising 9514 insertions, 8871 deletions, 60 inversions, and 3315 duplications (Table S6). The total length of the SVs spanned approximately 12.9 Mbp, accounting for roughly 0.4% of the genome. Notably, most SVs (90.53%) are smaller than 1 Kbp, reflecting a similar trend observed with ZF1 SVs (He et al. 2020) (Table S6). Among the 21,760 SVs in VHG, 20,400 SVs, accounting for 93.75%, were assigned to 22 chromosomes (including X and Y chromosomes), with no inversion found on chromosomes 13, 15, 18, 19, and 22 (Figure 1a). The maximum length of insertion, deletion, inversion, and duplication SVs were 15,835, 76,914, 90,932, and 681,903 bp, respectively (Figure 1b, Table S6). The median lengths of insertion, deletion, inversion, and duplication SVs, excluding outliers, were 244, 128, 1567, and 103 bp, respectively (Table S6, Figure 1c,d). The differences in the numbers of VHG SVs also can be compared with the 18,210, 17,900, 20,175, and 17,936/17,687 SVs detected in the AK1, ZF1, HX1, and Swe1/2 genomes, respectively [21,26,28,47]. Our findings indicated an equivalent analysis in detecting structural variants compared to previous studies on similar PacBio long-read datasets, which reported structural variant variation across various populations and highlighted the sensitivity of long-read sequencing technologies employed in research [21]. Additionally, the diversity and abundance of SVs observed in the VHG Kinh genome emphasize the genetic uniqueness of the Vietnamese population. This is in line with other recent genomic studies that underscore the importance of including diverse ethnic groups to capture the full spectrum of human genetic variation. This comprehensive catalog of SVs not only enhances our understanding of the Kinh Vietnamese genome but also contributes to the broader field of human genomics, providing valuable insights into the genetic diversity and structural complexity present within Southeast Asian populations.

Furthermore, an analysis of the distribution of SVs across the 24 chromosomes revealed a strong correlation between the length of our assembled chromosome and the number of SVs (R2 = 0.83, P = 4.59 × 10^−5^) (Figure 1e). This pattern has also been observed in a previous study [47].

### 3.3. Polishing and Quality Evaluation of the De Novo Assembly

**Figure 2:**
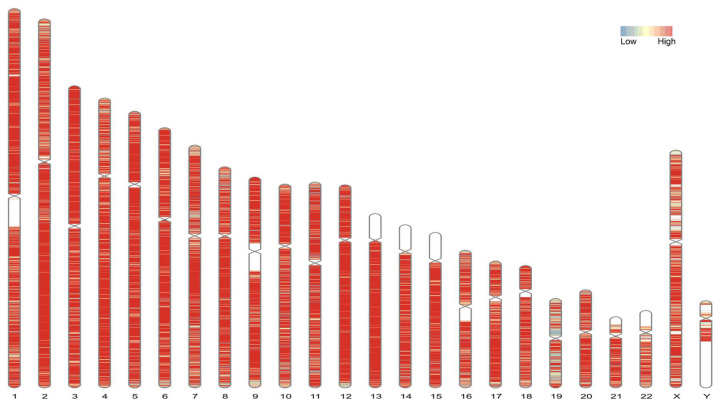
De novo assembly of the VHG genome compared to hg38. Super scaffolds in VHG1.1 are assigned to hg38 based on alignment data set by Lastz and visualization by KaryotypeR. The color segments present low to high percentage sequence identity of VHG1.1 sequence aligning to hg38 reference. The absence sequences are labeled by the white segments on each chromosome.

The hybrid super scaffold assembly was evaluated and corrected using Inspector to generate higher-quality contigs, removing misassembles and false structural variants (Figure 2). This process improved completeness, as measured by BUSCO score from 91.5% to 92, and reduced the number of small-scale assembly errors per Mbp decreased from 18.2 to 2.44. The corrected assembly, named VHG1.1, had a quality value (QV) evaluated by Inspector was 48.45%, corresponding to an accuracy of 99.9% and high contiguity with an N50 of 50.627 Mbp (>23 Mbp) [47,59] (Table S7).

To avoid the idiosyncratic nature of using the sequence of a single individual as a population-specific representative, we aimed to generate a more population-reflective reference. A typical KHV genome can differ from the reference human genome by 4 to 4.2 million sites; however, among these variants, around 40,000 are common (>5%) within the Kinh Vietnamese population [10]. In this study, the VHG1.1 assembly was “corrected” to VHG1.2 by removing rare and uninformative homozygous variants where other KHV genomes had consensus, thus adjusting these positions to the major, “common” allele in the population. JASPER polishing did not alter the assembly’s contiguity values; however, it increased the number of misaligned sequences to hg38 in VHG1.2 compared to VHG1.1.

Throughout the polishing process, all assemblies maintained a total genome length of 3.22 Gbp, closely approximating the expected size of the hg38 reference genome (3.21 Gbp). The longest scaffold length remained consistent across the assemblies, at approximately 145.9 Mbp, reflecting stability in long scaffold generation. The N50 values, a measure of assembly continuity, ranged from 50.63 to 50.64 Mbp across the assemblies, with minimal variation between them (Table 2, Table S8). 99.8% of contigs were mapped to scaffolds, and 95% of super scaffolds (283/295) were mapped to the 24 chromosomes. The size of each chromosome super scaffold and the unknown sequence are summarized in Table S9.

Comparing the final assembly VHG1.2 to the hg38 and T2T references showed high (>75%) identity scores of 86.3% and 92.1%, respectively (Table 2, Table S10, Figure S5). Notably, 12 super scaffolds totaling 8.78 Mbp failed to align with all references. Similar results were observed in previous publications, where the unaligned sequences ranged from 10.3 Mbp to 13.8 Mbp [1,28], and even in hg38 with 11.5 Mbp of unplaced and unlocalized sequences [16]. This improved genomic alignment when the T2T reference indicated that the VHG1.2 assembly, despite being incomplete, contains sequences not resolved in hg38. Interestingly, the VHG1.1 assembly showed no significant difference in matching with the T2T compared to hg38, suggesting that the extra polishing step not only corrected the assembly to a KHV consensus but also improved its completeness. This improvement was not reflected through the BUSCO score as this method only tested completeness using a set of conserved genes. We hypothesized that these changes in alignment were in genomic regions where the Illumina reads combined with PacBio HiFi reads reached a reliable threshold for JASPER to correct, and these regions were covered in T2T but not hg38. It was also possible that in hg38, these regions contained large-scale polymorphisms from the various contributing donors, whereas both T2T and VHG comprised only one donor’s genome or one fairly homogeneous population’s genome like the Kinh Vietnamese [16,60].

### 3.4. Toward the First Version of the Kinh Vietnamese Population-Specific Reference Genome

To validate VHG1.2 as a population-specific assembly for the Kinh Vietnamese, we used three additional KHV genomes from 1KGP we selected for variant calling analysis. The variant callers used in this study have been previously evaluated, tested, and benchmarked and are widely adopted in clinical genetics [61,62,63].

**Figure 3:**
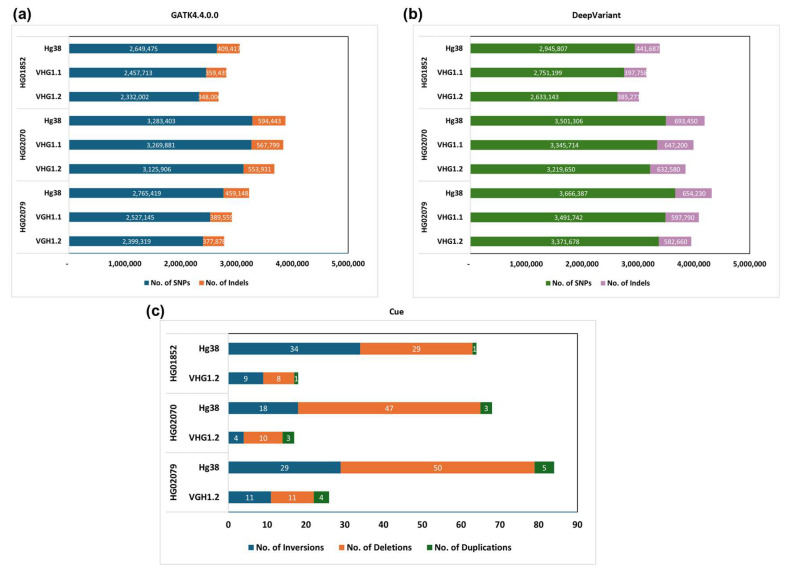
The number of variants discovered across three Vietnamese genomes from 1KGP based on VHG assemblies and hg38 references. (**a**) SNP and indel were detected by GATK 4.4.0.0. The dark teal and orange bars indicate the SNP and indel calls, respectively. (**b**) SNP and indel were detected by Deepvariant. The dark green and purple bars indicate the SNP and indel calls, respectively. (**c**) Structural variants ≥5 Kbp were detected by Cue. The dark teal, orange, and dark green bars indicate the inversion, deletion, and duplication calls, respectively.

We observed a similar trend across the output of different methods, where the number of SNPs and indels called was reduced when a VHG assembly was used as the reference compared to the hg38 reference (Figure 3a,b, Table S11). This result aligned with previous comparisons of the Han Chinese CN1 and HX1 reference genomes with T2T-CHM13 and hg38, respectively, in variant calling for samples of Chinese descent [20,21], as well as variant calling results using the Korean genome reference KOREF [24]. Polishing our assembly with other KHV genomes also affected the number of called variants, potentially reducing false negatives by using a more population-specific reference. In particular, as the reference instead of hg38, the de novo assembly VHG1.1 reduced the amount of SNP and indel calls by 800,000, and the population-specific VHG1.2 assembly reduced this number by a further 100,000 (approximately 3% of the total called SNPs and indels).

Interestingly, there was a significant difference in SNV calling results between GATK and DeepVariant, with GATK detecting up to 30% fewer SNPs and 15% fewer indels than DeepVariant. This discrepancy is likely due to the limitations of GATK, which make it more sensitive to artifacts and data quality issues, particularly sequencing depth and read mapping ambiguity [17,61].

DeepVariant, on the other hand, has been shown to perform better with low-coverage Illumina datasets of 12X or less [7,61], which applied to our test set. Despite these differences, both methods still showed a statistically significant reduction in the number of called SNPs and indels when VHG1.2 was used as a reference instead of hg38 (*p* < 0.05, see Table S12).

In addition, we utilized the test set for structural variant (SV) detection using Cue, a deep-learning-based variant caller. SV discovery, using only short-read Illumina data, remains highly challenging due to limited long-range contiguity [64]. However, despite being restricted to detecting only three types of SVs (deletions, inversion, and duplication) of 5 + Kbp or larger, Cue has demonstrated state-of-the-art performance among short-read callers, achieving high F1 scores even with low-coverage datasets [55]. By calling SVs in our test set using Cue, we were able to obtain highly confident estimates of the number of SVs per sample, as well as the number of SVs shared between individuals, enabling us to assess the specificity of our assembly in comparison with hg38 as a reference (Figure 3c, Table S13).

For large SVs greater than 2 Kbp, approximately 70% were shared across most super-populations, with 31% identified as unique to East Asian populations [65]. Previous studies also reported thousands of superfluous SVs in worldwide samples that most likely come from the lack of population-specific sequences in the hg19 and hg38 [34,66], as East Asian haplotypes only account for 4.3% of the Genome Reference Consortium’s reference genomes. Furthermore, Takayama et al. suggested that most of the Japanese population shared many of the identified SVs [34]. Based on this, we expected a significantly smaller number of SVs to be detected when using VHG1.2 as a reference compared to hg38. Indeed, Cue identified 3 to 4 times fewer SVs using VHG1.2, compared to hg38, mostly of deletion and inversion types (Figure 3c). The amount of shared SVs between all three individuals with hg38 as reference was 11.5% of the total SVs detected, but between each pair of samples, the amount of shared SVs reached up to 45.5%. In contrast, with VHG1.2 as a reference, only one SV (2.2%) was shared between all samples, and the amount of shared SVs between each pair of samples was approximately 25% (Table S13). Unfortunately, as we currently not yet be able to do this analysis with matching coordination between the two references, we could not ascertain how many of the called SVs were shared between hg38 and VHG1.2. This will be another aspect to look into when VHG1.2 can be completed and annotated by using higher coverage Illumina KHV datasets and Oxford Nanopore Technology (ONT) reads in the future, as it is another indicator of how accurate our assembly was compared to hg38.

### 3.5. Representativeness of VHG1.2 for the Vietnamese Population

**Figure 4:**
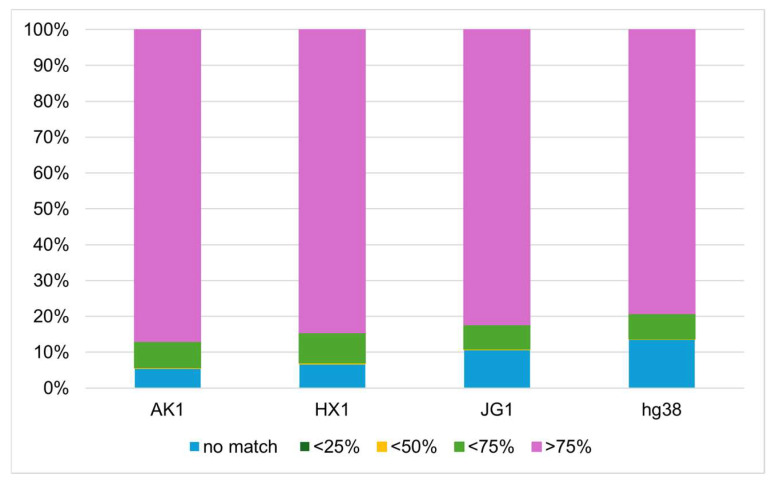
Proportion of sequence aligning between VHG1.2 and other assemblies.

Genome-to-genome alignments revealed that VHG1.2 shares a high degree of similarity with East Asian assemblies, with over 75% identity observed in AK1, HX1, and JG1 as 86.95%, 84.58%, and 82.29%, respectively. The mapping data show the greatest divergence between VHG1.2 and hg38, with only 79.25% of the sequence aligning at >75% identity (Figure 4 and Figure S6, Table S14). 

Interestingly, among the East Asian references, JG1 exhibited the most distant alignment with VHG1.2. This is consistent with previous findings that, although JG1 is of East Asian origin, it tends to localize outside the main East Asian cluster in population structure analyses [38]. Although VHG1.2 is still in its early version and has several limitations, these findings collectively support its representativeness for the Vietnamese population and its close genetic relationship with other East Asian populations.

## 4. Conclusions

VHG1.2 can be considered the highest-quality Kinh Vietnamese genome assembly to date with high completeness, accuracy, and contiguity, comparable to other high-quality population-specific de novo genome assemblies. This marks an important first step toward constructing a Vietnamese reference genome, which will facilitate the development of personalized medicine approaches in Vietnam. However, the VHG1.2 assembly is not entirely complete. This can be explained by the limitation of the sequencing data we produced and the capacity of our computational power.

## Figures and Tables

**Table 1:** Comparison of basic statistics with other high-quality de novo assemblies using the same method (PacBio + Bionano hybrid), with GRCh38 and T2T as references.

Assembly	Population	Sex	Total Length (Gbp)	Contigs		Hybrid Scaffold		Number of Gaps	N-Gap Length (Mbp)	Year	Reference
				Number	N50 (Mbp)	Number	N50 (Mbp)				
VHG1.2	Kinh Vietnamese	M	3.22	2198^1^	8.78^1^	295	50.6	-	-	2023	This study
JG1	Japanese	M	3.09	1068	23.6	624	142	473	2.51	2021	[39]
ZF1	Tibetan	M	2.9	3148 ^1^	23.6 ^1^	2321 ^2^	47.2 ^2^	740	7.82	2020	[47]
NH1.0	Northern Han Chinese	M	2.89	6663 ^1^	1.74 ^1^	5574 ^2^	46.6 ^2^	8484 ^2^	-	2019	[22]
Swe1	Swedish	M	3.13	3139 ^3^	9.47 ^3^	n/a	49.8	-	-	2018	[28]
Swe2	Swedish	F	3.1	3162 ^3^	8.52 ^3^	n/a	45.4	-	-	2018	[28]
AK1	Korean	M	2.9	4206	17.92	2832	44.9	264	3.74	2016	[26]
HX1	Chinese	M	2.93	5843	8.33	n/a	22.0	10,901	39.34	2016	[21]
HG001/NA12878	European	F	3.18	-	1.4	202	31.3	2332	146.4	2015	[38]
GRCh38.p14	Reference	M	3.21	996	57.9	470	67.8	349	150.6	2022	NCBI
T2T-CHM13v2.0	Reference	-	3.05	23	154.3	23	154.3	0	0	2022	[59]

“-” represents data not available. ^1^ PacBio assembly statistics. ^2^ Merged 10X linked read with PacBio + Bionano hybrid data. ^3^ Only primary contigs with a 20 Kbp cut-off.

**Table 2:** Evaluation of VHG genome assemblies through multiple parameters. Continuity was assessed with the number of scaffolds, contig length, and N50 values; accuracy was evaluated by the genome fraction that matches with standard references; and completeness was measured by BUSCO score.

Parameters	Super Scaffolds	VHG1.1	VHG1.2
Number of scaffolds	295	295	295
Total length	3,225,940,482	3,222,774,051	3,222,784,990
N50	50,640,186	50,627,973	50,628,337
GC (%)	40.85	40.85	40.85
Largest contig	145,919,952	145,820,377	145,820,867
>50% identity with hg38	90.58%	90.65%	86.26%
>75% identity with hg38	82.63%	83.93%	79.25%
>50% identity with T2T	90.19%	90.26%	92.06%
>75% identity with T2T	83.69%	82.90%	85.88%
BUSCO score (%)	91.5	92	92

## Data Availability

The HiFi Pacbio reads of VHG have been deposited on NCBI under the accession of BioProject: PRJNA1075075, BioSample: SAMN39902363, and SRA: SRR27928179. The final version of Kinh Vietnamese Reference Genome VHG1.2 has been deposited on the local server of the Vietnam Academy of Science and Technology (VAST). All command lines are stored on GitHub platform.

## Supplementary Materials

The following supporting information can be downloaded at https://www.mdpi.com/article/10.3390/genes16050536/s1: Figure S1: Blood Genomic DNA Analysis; Figure S2: Karyotype analysis of donor VHG; Figure S3: VHG mitochondrial genome (mtDNA) generated by HiFi reads using Hifiasm tools; Figure S4: The assembly gap in the super scaffold 546 was filled by integrating long-read sequencing and physical mapping; Figure S5: The dotplot represents the co-linearity between VHG1.2 and the reference genomes hg38 or T2T using D-GENIES, with the filter option applied to remove sequences with identity < 75%; Figure S6: The dotplot represents the co-linearity between VHG1.2 and other assemblies as hg38, Han Chinese (HX1), Korean (AK1), and Japanese (JG1) using D-GENIES with the filter option applied to remove sequences with identity < 75%; Table S1: Summary of the raw HiFi sequencing read data; Table S2: Summary of the CCS data; Table S3: Summary of the Bionano optical mapping data; Table S4: The characteristics of different versions of the VHG assemblies; Table S5: Comparison of the number of SNPs and indels identified in VHG and hg38; Table S6: Analysis of structural variants (SVs) detected in VHG; Table S7: Evaluation of the VHG1.1 genome assembly after error correction; Table S8: Assembly quality comparison of VHG1.1 and VHG1.2; Table S9: Arrangement of super-scaffolds into chromosomal-level scaffolds; Table S10: Summary of the VHG1.2 genome assemblies using Minimap2; Table S11: Comparison of the number of SNPs and indels in three Vietnamese genomes from the 1000 Genomes Project, using VHG and hg38 as reference genomes; Table S12: *P*-values from pairwise comparisons of the number of detected variants between different reference genomes; Table S13: Structural variant (SV) counts in three Vietnamese genomes from 1KGP, detected with Cue using VHG1.2 and GRCh38 as references; Table S14: Alignment summary of VHG1.2 with other assemblies (Han Chinese (HX1), Korean (AK1), and Japanese (JG1)) using Minimap2.

## Abbreviations

The following abbreviations are used in this manuscript:

WGS	Whole-genome sequences
Kbp	Kilobases
Mbp	Megabases
SNP	Single-nucleotide polymorphism
Indel	Short insertions–deletions
SV	Structure variant
JASPER	Jellyfish-based Assembly Sequence Polisher for Error Reduction
KHV	Kinh Vietnamese genome
ONT	Oxford Nanopore Technology
hg19	GRCh37
hg38	GRCh38

## References

[1] Hu T., Chitnis N., Monos D., Dinh A. (2021). Next-generation sequencing technologies: An overview. Hum. Immunol..

[2] Kim H.M., Jeon S., Chung O., Jun J.H., Kim H.S., Blazyte A., Lee H.Y., Yu Y., Cho Y.S., Bolser D.M. (2021). Comparative analysis of 7 short-read sequencing platforms using the Korean Reference Genome: MGI and Illumina sequencing benchmark for whole-genome sequencing. GigaScience.

[3] Conlin L.K., Aref-Eshghi E., McEldrew D.A., Luo M., Rajagopalan R. (2022). Long-read sequencing for molecular diagnostics in constitutional genetic disorders. Hum. Mutat..

[4] Logsdon G.A., Vollger M.R., Eichler E.E. (2020). Long-read human genome sequencing and its applications. Nat. Rev. Genet..

[5] National Research Council (2009). An Assessment of the SBIR Program at the National Institutes of Health.

[6] Kingsmore S.F., Nofsinger R., Ellsworth K. (2024). Rapid genomic sequencing for genetic disease diagnosis and therapy in intensive care units: A review. NPJ Genom. Med..

[7] Adams D.R., Eng C.M. (2018). Next-Generation Sequencing to Diagnose Suspected Genetic Disorders. N. Engl. J. Med..

[8] Nagasaki M., Kuroki Y., Shibata T.F., Katsuoka F., Mimori T., Kawai Y., Minegishi N., Hozawa A., Kuriyama S., Suzuki Y. (2019). Construction of JRG (Japanese reference genome) with single-molecule real-time sequencing. Hum. Genome Var..

[9] Chaisson M.J.P., Huddleston J., Dennis M.Y., Sudmant P.H., Malig M., Hormozdiari F., Antonacci F., Surti U., Sandstrom R., Boitano M. (2015). Resolving the complexity of the human genome using single-molecule sequencing. Nature.

[10] Auton A., Brooks L.D., Durbin R.M., Garrison E.P., Kang H.M., Korbel J.O., Marchini J.L., McCarthy S., McVean G.A., Abecasis G.R. (2015). A global reference for human genetic variation. Nature.

[11] Mak A.C.Y., Lai Y.Y.Y., Lam E.T., Kwok T.P., Leung A.K.Y., Poon A., Mostovoy Y., Hastie A.R., Stedman W., Anantharaman T. (2016). Genome-wide structural variation detection by genome mapping on nanochannel arrays. Genetics.

[12] Levy S., Sutton G., Ng P.C., Feuk L., Halpern A.L., Walenz B.P., Axelrod N., Huang J., Kirkness E.F., Denisov G. (2007). The diploid genome sequence of an individual human. PLoS Biol..

[13] Popejoy A.B., Fullerton S.M. (2016). Genomics is failing on diversity. Nature.

[14] Sirugo G., Williams S.M., Tishkoff S.A. (2019). The Missing Diversity in Human Genetic Studies. Cell.

[15] Ballouz S., Dobin A., Gillis J.A. (2019). Is it time to change the reference genome?. Genome Biol..

[16] Aganezov S., Yan S.M., Soto D.C., Kirsche M., Zarate S., Avdeyev P., Taylor D.J., Shafin K., Shumate A., Xiao C. (2022). A complete reference genome improves analysis of human genetic variation. Science.

[17] Barbitoff Y.A., Abasov R., Tvorogova V.E., Glotov A.S., Predeus A.V. (2022). Systematic benchmark of state-of-the-art variant calling pipelines identifies major factors affecting accuracy of coding sequence variant discovery. BMC Genom..

[18] Yang X., Lee W.P., Ye K., Lee C. (2019). One reference genome is not enough. Genome Biol..

[19] Thorburn D.J., Sagonas K., Binzer-Panchal M., Chain F.J.J., Feulner P.G.D., Bornberg-Bauer E., Reusch T.B.H., Samonte-Padilla I.E., Milinski M., Lenz T.L. (2023). Origin matters: Using a local reference genome improves measures in population genomics. Mol. Ecol. Resour..

[20] Yang C., Zhou Y., Song Y., Wu D., Zeng Y., Nie L., Liu P., Zhang S., Chen G., Xu J. (2023). The complete and fully-phased diploid genome of a male Han Chinese. Cell Res..

[21] Shi L., Guo Y., Dong C., Huddleston J., Yang H., Han X., Fu A., Li Q., Li N., Gong S. (2016). Long-read sequencing and de novo assembly of a Chinese genome. Nat. Commun..

[22] Du Z., Ma L., Qu H., Chen W., Zhang B., Lu X., Zhai W., Sheng X., Sun Y., Li W. (2019). Whole Genome Analyses of Chinese Population and De Novo Assembly of A Northern Han Genome. Genom. Proteom. Bioinform..

[23] Yang X., Zhao X., Qu S., Jia P., Wang B., Gao S., Xu T., Zhang W., Huang J., Ye K. (2022). Haplotype-resolved Chinese male genome assembly based on high-fidelity sequencing. Fundam. Res..

[24] Cho Y.S., Kim H., Kim H.M., Jho S., Jun J., Lee Y.J., Chae K.S., Kim C.G., Kim S., Eriksson A. (2016). An ethnically relevant consensus Korean reference genome is a step towards personal reference genomes. Nat. Commun..

[25] Seo J.S., Rhie A., Kim J., Lee S., Sohn M.H., Kim C.U., Hastie A., Cao H., Yun J.Y., Kim J. (2016). De novo assembly and phasing of a Korean human genome. Nature.

[26] Maretty L., Jensen J.M., Petersen B., Sibbesen J.A., Liu S., Villesen P., Skov L., Belling K., Theil Have C., Izarzugaza J.M.G. (2017). Sequencing and de novo assembly of 150 genomes from Denmark as a population reference. Nature.

[27] Ameur A., Che H., Martin M., Bunikis I., Dahlberg J., Höijer I., Häggqvist S., Vezzi F., Nordlund J., Olason P. (2018). De novo assembly of two Swedish genomes reveals missing segments from the human GRCh38 reference and improves variant calling of population-scale sequencing data. Genes.

[28] Takayama J. (2024). Technical Notes on the Construction of the Japanese Near T2T Reference Genome, JG3. Japanese Multi-Omics Reference Panel Portal. https://jmorp.megabank.tohoku.ac.jp/downloads/tommo-jg3.0.0-20240618.

[29] Kim H.S., Jeon S., Kim C., Kim Y.K., Cho Y.S., Kim J., Blazyte A., Manica A., Lee S., Bhak J. (2019). Chromosome-scale assembly comparison of the Korean Reference Genome KOREF from PromethION and PacBio with Hi-C mapping information. GigaScience.

[30] Chao K.H., Zimin A.V., Pertea M., Salzberg S.L. (2023). The first gapless, reference-quality, fully annotated genome from a Southern Han Chinese individual. G3 Genes Genomes Genet..

[31] Sriwichaiin S., Makino S., Funayama T., Otsuki A., Kawashima J., Okamura Y., Tadaka S., Katsuoka F., Kumada K., The Tohoku Medical Megabank Project Study Group (2024). JG2: An updated version of the Japanese population-specific reference genome. bioRxiv.

[32] Hwang M.Y., Choi N.H., Won H.H., Kim B.J., Kim Y.J. (2022). Analyzing the Korean reference genome with meta-imputation increased the imputation accuracy and spectrum of rare variants in the Korean population. Front. Genet..

[33] Amarasinghe S.L., Su S., Dong X., Zappia L., Ritchie M.E., Gouil Q. (2020). Opportunities and challenges in long-read sequencing data analysis. Genome Biol..

[34] Rhoads A., Au K.F. (2015). PacBio Sequencing and Its Applications. Genom. Proteom. Bioinform..

[35] Jain M., Koren S., Miga K.H., Quick J., Rand A.C., Sasani T.A., Tyson J.R., Beggs A.D., Dilthey A.T., Fiddes I.T. (2018). Nanopore sequencing and assembly of a human genome with ultra-long reads. Nat. Biotechnol..

[36] Lin B., Hui J., Mao H. (2021). Nanopore technology and its applications in gene sequencing. Biosensors.

[37] Yuan Y., Chung C.Y., Chan T.F. (2020). Advances in optical mapping for genomic research. Comput. Struct. Biotechnol. J..

[38] Takayama J., Tadaka S., Yano K., Katsuoka F., Gocho C., Funayama T., Makino S., Okamura Y., Kikuchi A., Sugimoto S. (2021). Construction and integration of three de novo Japanese human genome assemblies toward a population-specific reference. Nat. Commun..

[39] ten Berk de Boer E., Ameur A., Bunikis I., Ek M., Stattin E.-L., Feuk L., Eisfeldt J., Lindstrand A. (2024). Long-read sequencing and optical mapping generates near T2T assemblies that resolves a centromeric translocation. Sci. Rep..

[40] Pischedda S., Barral-Arca R., Gómez-Carballa A., Pardo-Seco J., Catelli M.L., Álvarez-Iglesias V., Cárdenas J.M., Nguyen N.D., Ha H.H., Le A.T. (2017). Phylogeographic and genome-wide investigations of Vietnam ethnic groups reveal signatures of complex historical demographic movements. Sci. Rep..

[41] Hai D.T., Thanh N.D., Trang P.T.M., Quang L.S., Hang P.T.T., Cuong D.C., Phuc H.K., Duc N.H., Dong D.D., Minh B.Q. (2015). Whole genome analysis of a Vietnamese trio. J. Biosci..

[42] Lischer H.E.L., Shimizu K.K. (2017). Reference-guided de novo assembly approach improves genome reconstruction for related species. BMC Bioinform..

[43] Kim J.I., Ju Y.S., Park H., Kim S., Lee S., Yi J.H., Mudge J., Miller N.A., Hong D., Bell C.J. (2009). A highly annotated whole-genome sequence of a Korean individual. Nature.

[44] Cheng H., Concepcion G.T., Feng X., Zhang H., Li H. (2021). Haplotype-resolved de novo assembly using phased assembly graphs with HiFiasm. Nat. Methods.

[45] Garg S., Fungtammasan A., Carroll A., Chou M., Schmitt A., Zhou X., Mac S., Peluso P., Hatas E., Ghurye J. (2021). Chromosome-scale, haplotype-resolved assembly of human genomes. Nat. Biotechnol..

[46] He Y., Lou H., Cui C., Deng L., Gao Y., Zheng W., Guo Y., Wang X., Ning Z., Li J. (2020). De novo assembly of a Tibetan genome and identification of novel structural variants associated with high-altitude adaptation. Natl. Sci. Rev..

[47] Chen Y., Zhang Y., Wang A.Y., Gao M., Chong Z. (2021). Accurate long-read de novo assembly evaluation with Inspector. Genome Biol..

[48] Li H. (2018). Minimap2: Pairwise alignment for nucleotide sequences. Bioinformatics.

[49] Alonge M., Soyk S., Ramakrishnan S., Wang X., Goodwin S., Sedlazeck F.J., Lippman Z.B., Schatz M.C. (2019). RaGOO: Fast and accurate reference-guided scaffolding of draft genomes. Genome Biol..

[50] Harris R.S. (2007). Improved Pairwise Alignment of Genomic DNA. https://api.semanticscholar.org/CorpusID:18002845.

[51] Cabanettes F., Klopp C. (2018). D-GENIES: Dot plot large genomes in an interactive, efficient and simple way. PeerJ.

[52] Simão F.A., Waterhouse R.M., Ioannidis P., Kriventseva E.V., Zdobnov E.M. (2015). BUSCO: Assessing genome assembly and annotation completeness with single-copy orthologs. Bioinformatics.

[53] Xie H., Li W., Hu Y., Yang C., Lu J., Guo Y., Wen L., Tang F. (2022). De novo assembly of human genome at single-cell levels. Nucleic Acids Res..

[54] Zheng-Bradley X., Streeter I., Fairley S., Richardson D., Clarke L., Flicek P. (2017). Alignment of 1000 Genomes Project reads to reference assembly GRCh38. GigaScience.

[55] Li H. (2013). Aligning sequence reads, clone sequences and assembly contigs with BWA-MEM. arXiv.

[56] Broad Institute (2024). GATK Documentation. https://gatk.broadinstitute.org/hc/en-us/community/posts/26880110839707-gatk-CombineGVCFs-output-contains-only-one-Chr.

[57] Poplin R., Chang P.C., Alexander D., Schwartz S., Colthurst T., Ku A., Newburger D., Dijamco J., Nguyen N., Afshar P.T. (2018). A universal SNP and small-indel variant caller using deep neural networks. Nat. Biotechnol..

[58] Popic V., Rohlicek C., Cunial F., Hajirasouliha I., Meleshko D., Garimella K., Maheshwari A. (2023). Cue: A deep-learning framework for structural variant discovery and genotyping. Nat. Methods.

[59] Nurk S., Koren S., Rhie A., Rautiainen M., Bzikadze A.V., Mikheenko A., Vollger M.R., Altemose N., Uralsky L., Gershman A. (2022). The complete sequence of a human genome. Science.

[60] Vu-Trieu A., Djoulah S., Tran-Thi C., Ngyuyen-Thanh T., Le Monnier De Gouville I., Hors J., Sanchez-Mazas A. (1997). HLA-DR and –DQB1 DNA polymorphisms in a Vietnamese Kinh population from Hanoi. Eur. J. Immunogenet..

[61] Betschart R.O., Thiéry A., Aguilera-Garcia D., Zoche M., Moch H., Twerenbold R., Zeller T., Blankenberg S., Ziegler A. (2022). Comparison of calling pipelines for whole genome sequencing: An empirical study demonstrating the importance of mapping and alignment. Sci. Rep..

[62] Lin Y.L., Chang P.C., Hsu C., Hung M.Z., Chien Y.H., Hwu W.L., Lai F., Lee N.C. (2022). Comparison of GATK and DeepVariant by trio sequencing. Sci. Rep..

[63] Chen N.C., Kolesnikov A., Goel S., Yun T., Chang P.C., Carroll A. (2023). Improving variant calling using population data and deep learning. BMC Bioinform..

[64] Zhang L., Zhou X., Weng Z., Sidow A. (2019). De novo diploid genome assembly for genome-wide structural variant detection. NAR Genom. Bioinform..

[65] Levy-Sakin M., Pastor S., Mostovoy Y., Li L., Leung A.K.Y., McCaffrey J., Young E., Lam E.T., Hastie A.R., Wong K.H.Y. (2019). Genome maps across 26 human populations reveal population-specific patterns of structural variation. Nat. Commun..

[66] Wu Z., Jiang Z., Li T., Xie C., Zhao L., Yang J., Ouyang S., Liu Y., Li T., Xie Z. (2021). Structural variants in the Chinese population and their impact on phenotypes, diseases and population adaptation. Nat. Commun..

