# Supplementary material for: Toward a Kinh Vietnamese Reference Genome: Constructing a De Novo Genome Assembly Using Long-Read Sequencing and Optical Mapping
Source: Genes (Basel). 2025 Apr 29;16(5):536. doi: 10.3390/genes16050536 (PMC12111184; doi:10.3390/genes16050536)
Supplement: Supplementary file 1 [file genes-16-00536-s001.zip › Supplementary Figure.pdf]

Towards a Kinh Vietnamese Reference Genome: Constructing a De Novo Genome Assembly Using Long-Read Sequencing and Optical Mapping

Le Thi Dung[1,2], Le Tung Lam[1], Nguyen Hong Trang[1,3], Nguyen Vu Hung Anh[1], Nguyen Ngoc Nam[1], Doan Thi Nhung[1], Tran Huyen Linh[1,2], Le Ngoc Giang[4], Hoang Ha[1], Nguyen Quang Huy[2], Truong Nam Hai[1,5]*

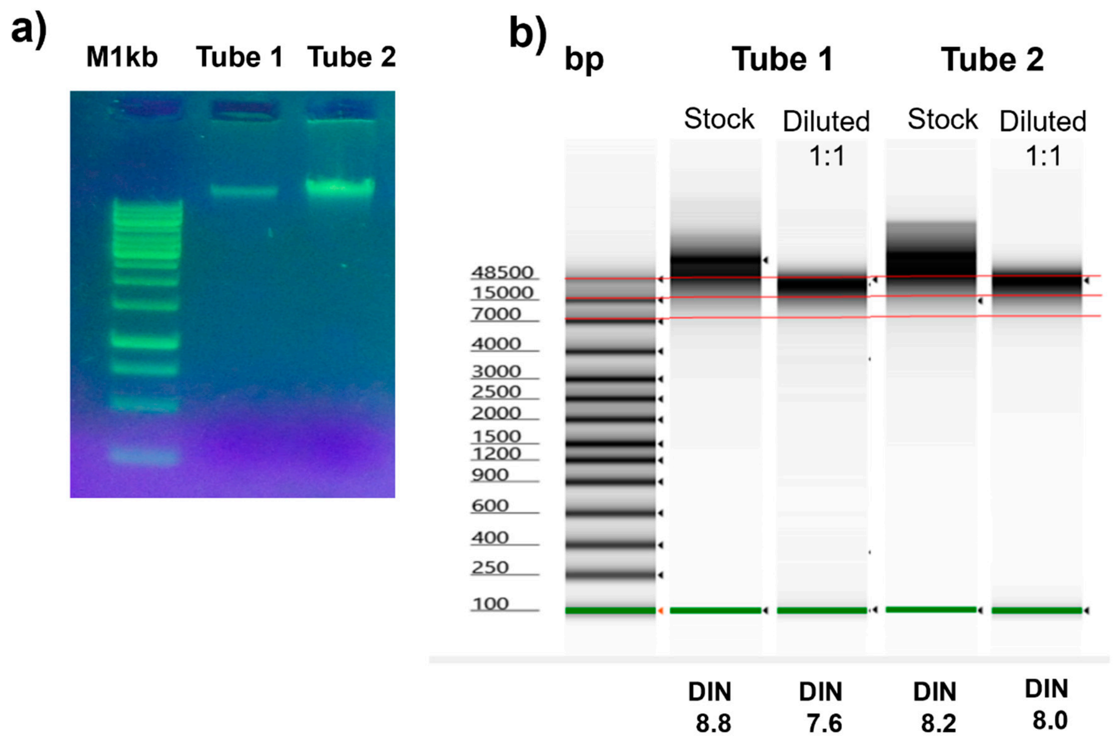

Figure S1. Blood Genomic DNA Analysis. a) High-molecular-weight genomic DNA was extracted from fresh blood using the Blood & Cell Culture DNA Midi Kit (QIAGEN, Germany). b) The genomic DNA quality was assessed using the Agilent 2200 TapeStation System. DIN: DNA Integrity Number.

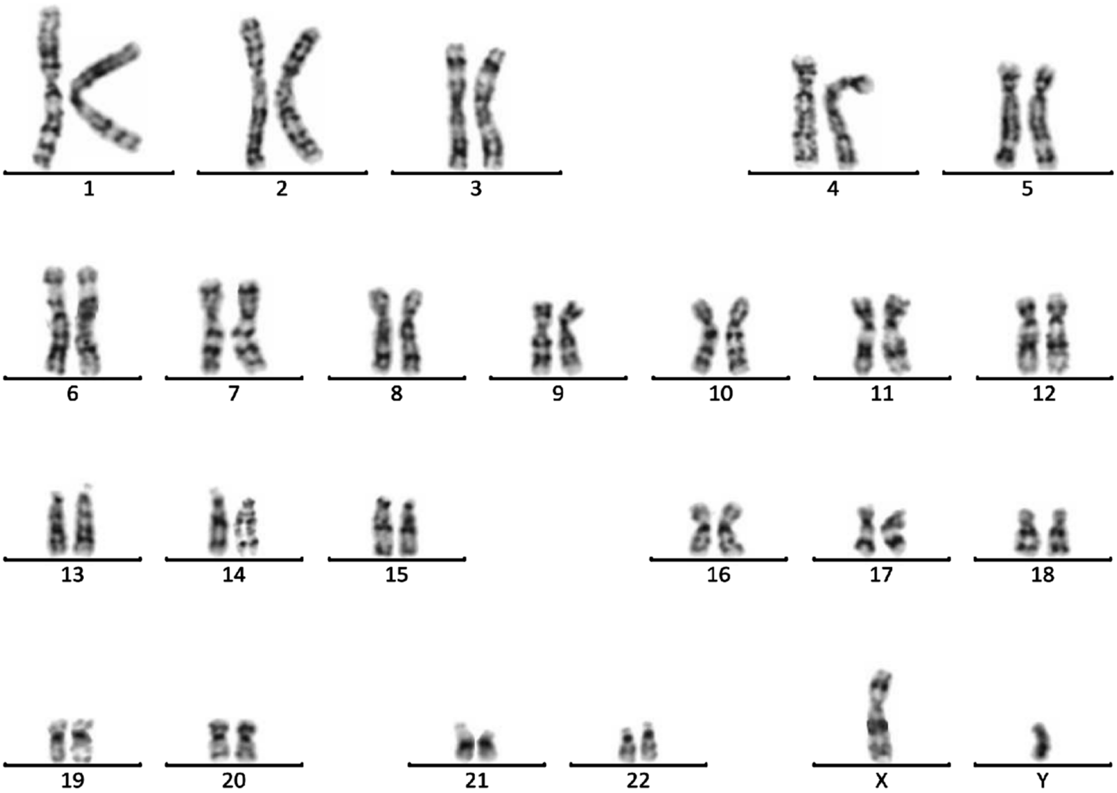

Figure S2. Karyotype analysis of donor VHG. Chromosome analysis was performed at a resolution of 450 bands. A total of 20 cells were observed, all showing a normal human male with 46 chromosomes, including X and Y. Additionally, five cells were analyzed for length and chromosomal abnormalities.

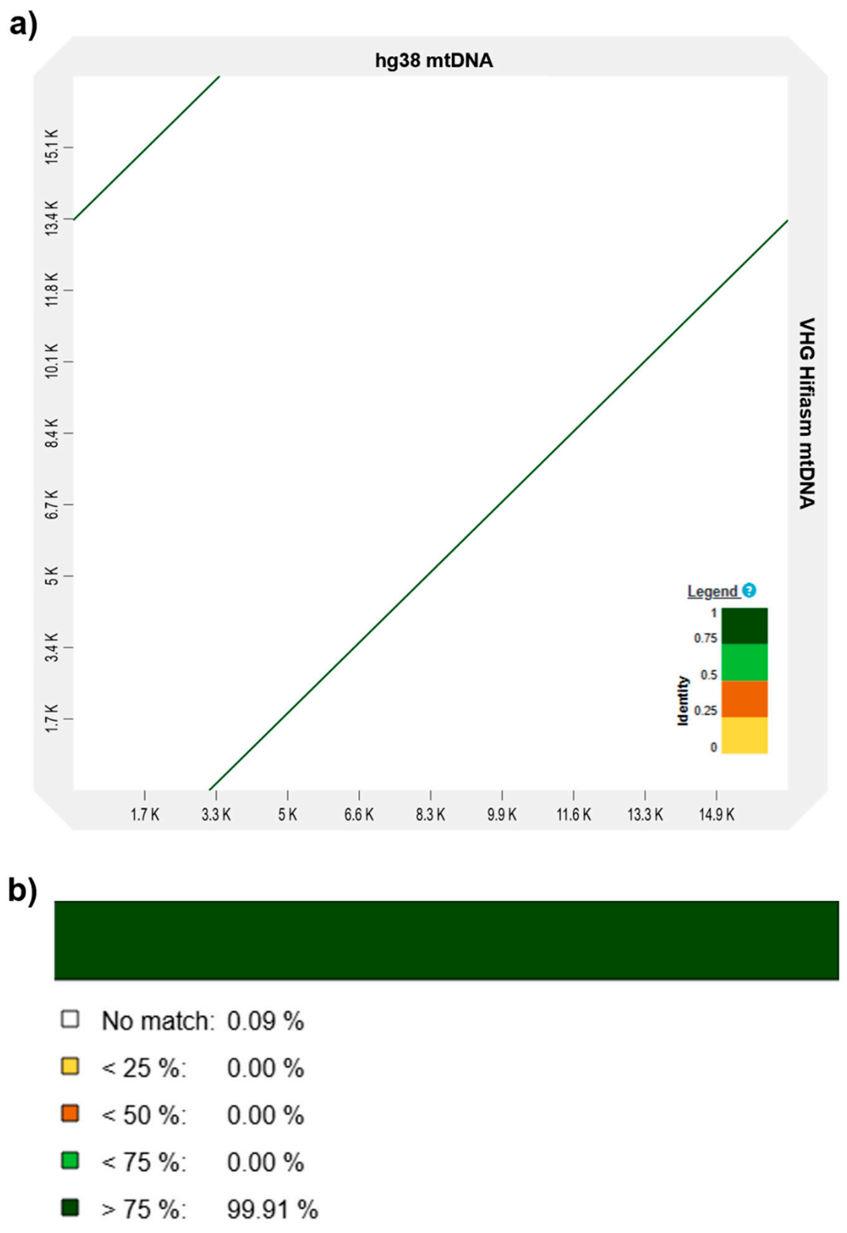

Figure S3. VHG mitochondrial genome (mtDNA) generated by HiFi reads using Hifiasm tools. a) The dotplot represents the co-linearity between VHG mtDNA and hg38 mtDNA using D-GENIES. Dark green indicates high identity (≥ 0.75 or 75%). b) Summary of VHG mtDNA sequence aligned with hg38 mtDNA using Minimap2.

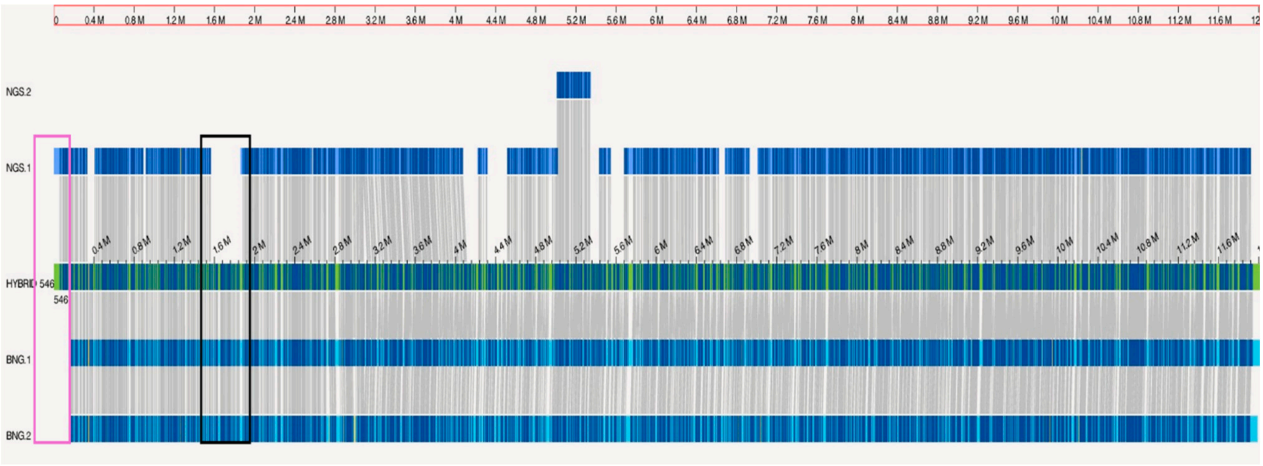

Figure S4. The assembly gap in the super scaffold 546 was filled by integrating long-read sequencing and physical mapping. NGS.1 and NGS.2 represent data from next-generation sequencing runs. BNG.1 and BNG.2 are Bionano genome (BNG) optical maps from two datasets or replicates. The grey lines indicate an alignment between labels on NGS, BNG and Hybrid scaffold. HYBRID 546 represents the hybrid scaffold that integrates NGS contigs (Hifi contigs) and Bionano optical maps. Blue bands in the hybrid scaffold show the optical map data, representing label locations detected during optical mapping. Purple and black boxes highlight areas without data in BNG and NGS, respectively.

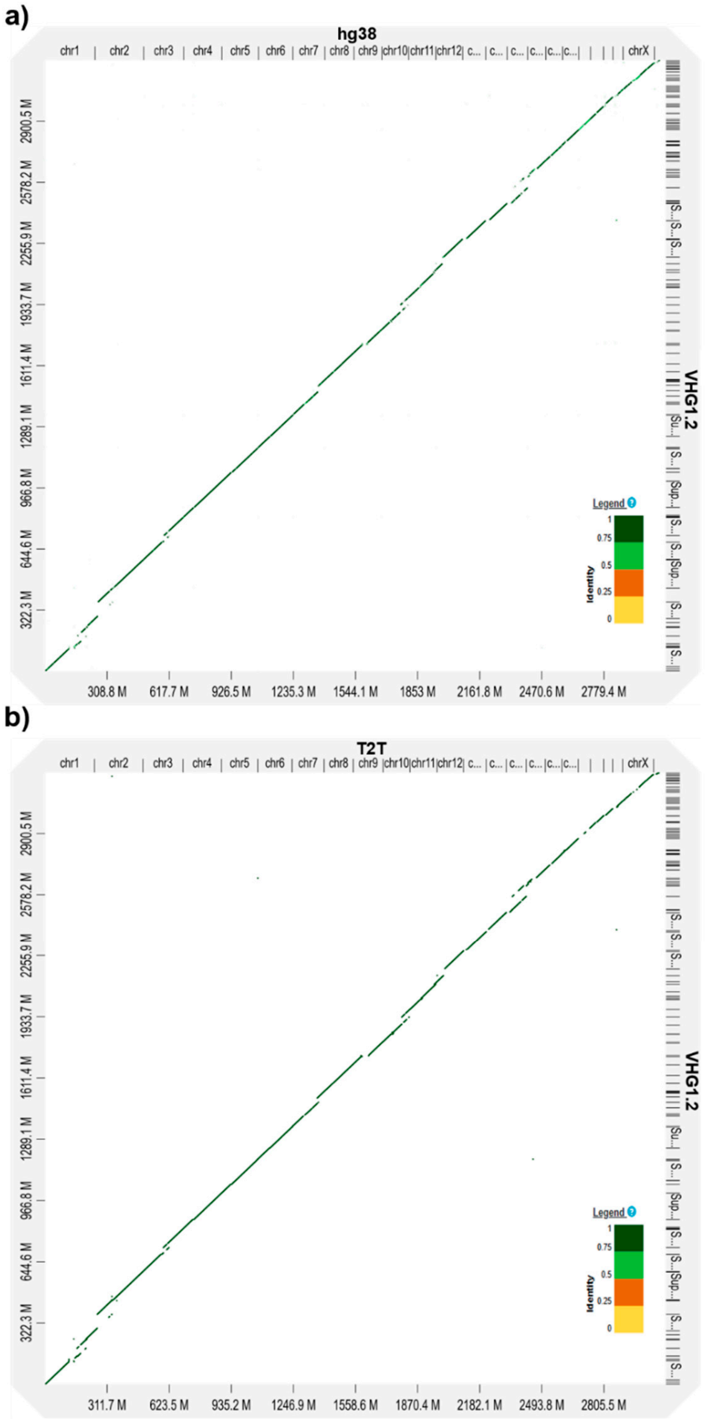

Figure S5: The dotplot represents the co-linearity between VHG1.2 and the reference genomes hg38 or T2T using D-GENIES, with the filter option applied to remove sequences with identity < 75%.

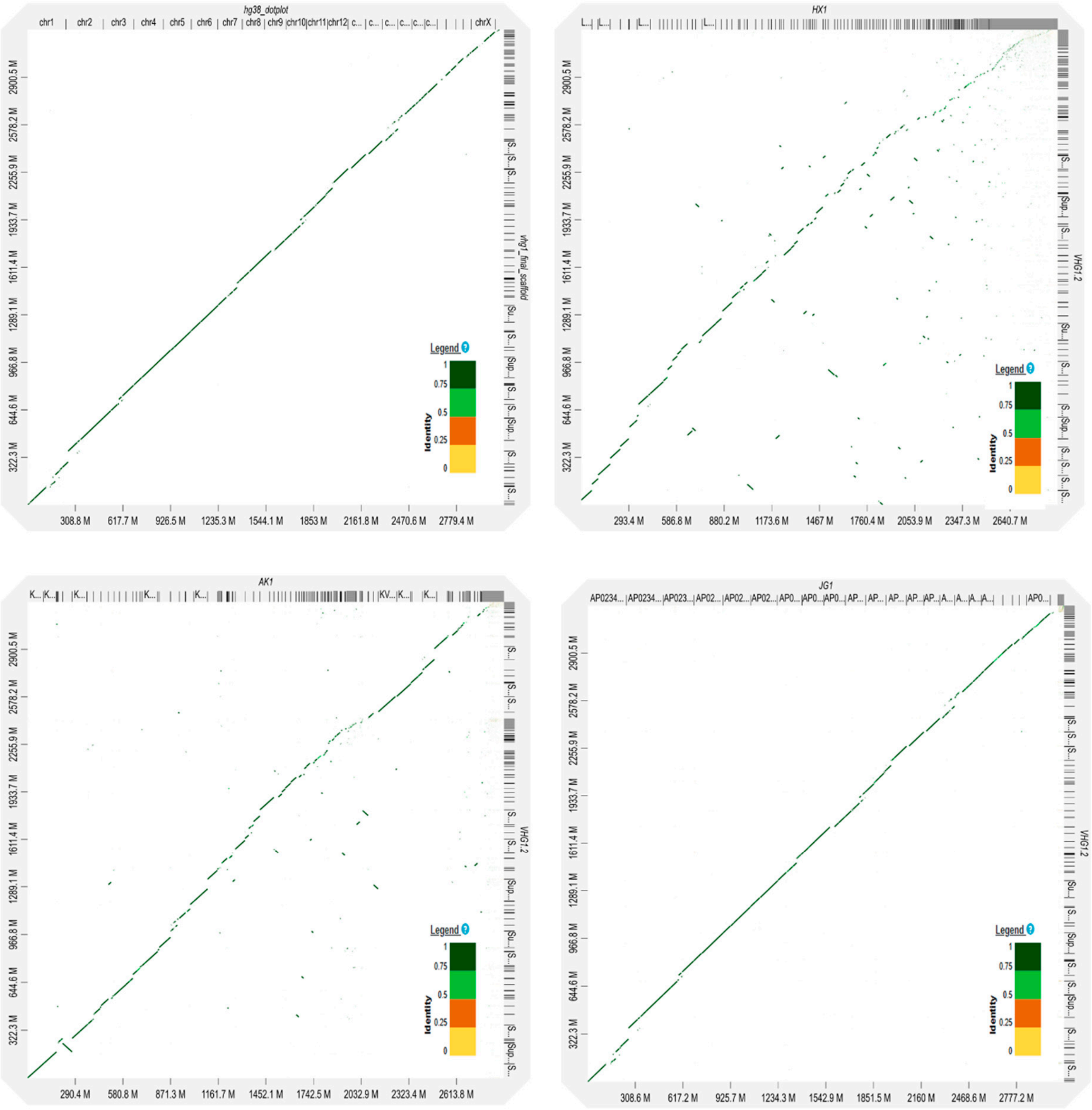

Figure S6. The dotplot represents the co-linearity between VHG1.2 and other assemblies as hg38, Han Chinese (HX1), Korean (AK1), and Japanese (JG1) using D-GENIES with the filter option applied to remove sequences with identity < 75%.
