# Supplementary material for: Toward a Kinh Vietnamese Reference Genome: Constructing a De Novo Genome Assembly Using Long-Read Sequencing and Optical Mapping
Source: Genes (Basel). 2025 Apr 29;16(5):536. doi: 10.3390/genes16050536 (PMC12111184; doi:10.3390/genes16050536)
Supplement: Supplementary file 1 [file genes-16-00536-s001.zip › Supplementary Table.pdf]

Towards a Kinh Vietnamese Reference Genome: Constructing a De Novo Genome Assembly Using Long-Read Sequencing and Optical Mapping

Le Thi Dung[1,2], Le Tung Lam[1], Nguyen Hong Trang[1,3], Nguyen Vu Hung Anh[1], Nguyen Ngoc Nam[1], Doan Thi Nhung[1], Tran Huyen Linh[1,2], Le Ngoc Giang[4], Hoang Ha[1], Nguyen Quang Huy[2], Truong Nam Hai[1,5*]

Table S1. Summary of the raw HiFi sequencing read data

No.	Parameter	Length (bp)
1	Polymerase read bases	694,388,307,937
2	Polymerase read	11,092,192
3	Polymerase read length (mean)	112,489
4	Polymerase read N50	232,929
5	Subread length (mean)	10,292
6	Subread N50	12,190
7	Longest subread length (mean)	16,804
8	Longest subread N50	20,030
9	Unique Molecular Yield	160,867,991,552

Table S2. Summary of the CCS data

No.	Parameter	Value
1	Hifi reads	5,284,005
2	Hifi yield (bp)	61,916,328,149
3	Hifi read length (mean, bp)	11,727
4	Hifi read quality (median)	Q36

Table S3. Summary of the Bionano optical mapping data

No.	Parameter	Raw data	Filtering data
1	Total number molecular	1,841,668	1,379,620
2	Total length (Mbp)	400,102.09	321,200.99
3	Average length (kbp)	217.25	232.82
4	N50 molecular (kbp)	205.5	219.56
5	Marker density (kbp)	15.77 /100	15.12 /100
6	Coverage compared to the reference genome	129.57 X	104.01 X

Table S4. The characteristics of different versions of the VHG assemblies

No.	Parameter	Hifiasm assembly	BioNano assembly	Super scaffolds	VHG1.1	VHG1.2
1	Contigs/Scaffolds/Super Scaffolds	2,198	1,042	295	295	295
2	N50 length (Mbp)	8.78	39.14	50.64	50.62	50.63
3	Max length (Mbp)	78.82	108.00	145.92	145.82	145.82
4	Total length (Mbp)	3,014.71	5,840.21	3,225.94	3,222.77	3,222.78

Table S5. Comparison of the number of SNPs and indels identified in VHG and hg38

Chromosome	No. of SNPs	No. of indels							
		**Length**	**1-3bp**	**4-6bp**	**7-10bp**	**11-30bp**	**31-50bp**	**>50bp**	**Total**
chr1	306,342	113,338	98,508	7,787	2,939	3,402	419	283	226,676
chr10	194,918	68,759	59,102	5,016	1,907	2,229	283	222	137,518
chr11	178,695	62,190	53,984	4,327	1,663	1,829	232	155	124,380
chr12	173,920	67,241	58,374	4,649	1,766	1,997	264	191	134,482
chr13	145,197	48,547	41,606	3,658	1,402	1,493	214	174	97,094
chr14	133,410	44,647	38,657	3,195	1,176	1,347	151	121	89,294
chr15	106,721	38,532	33,435	2,723	1,018	1,131	119	106	77,064
chr16	116,431	41,534	35,852	2,898	1,096	1,347	191	150	83,068
chr17	100,731	42,760	37,321	2,904	1,044	1,216	163	112	85,520
chr18	108,749	36,775	31,634	2,708	1,026	1,127	157	123	73,550
chr19	75,829	35,715	31,132	2,391	888	1,024	152	128	71,430
chr2	304,373	111,692	96,649	7,845	3,038	3,391	447	322	223,384
chr20	107,929	33,580	29,135	2,200	871	1,108	141	125	67,160
chr21	72,698	20,605	17,521	1,552	557	729	114	132	41,210
chr22	68,115	21,868	18,762	1,561	613	712	109	111	43,736
chr3	261,546	92,356	80,158	6,492	2,542	2,650	278	236	184,712
chr4	273,892	89,276	76,850	6,632	2,361	2,810	336	287	178,552
chr5	225,499	78,416	68,275	5,328	1,994	2,338	267	214	156,832
chr6	224,022								
chr7	225,015								
chr8	187,233								
chr9	178,530								
chrX	82,564								
chrM	39								
chrUn	39,976								
chrY	13,211								

**Total**	**3,905,585**								**2,095,662**

Table S6. Analysis of structural variants (SVs) detected in VHG

SVs type	Total SVs detected in VHG			SVs assigned on chromosome		
	**SV length <1000 bp**	**SV length >=1000 bp**	**Total**	**No. of SV**	**Median length (bp)**	**Maximum length (bp)**
Deletion	8,042	829	8,871	8,159	128	76,914
Insertion	8,371	1,143	9,514	8,892	244	15,835
Duplication	3,263	52	3,315	3,134	103	681,903
Inversion	24	36	60	55	1,567	90,932
Total	19,700		21,760	20,400		
Total length (bp)			12,885,450			

Table S7. Evaluation of the VHG1.1 genome assembly after error correction

Parameters	Super scaffolds	VHG1.1
**Inspector**		
Number of scaffolds	295	295
Number of scaffolds > 10000 bp	295	295
Number of scaffolds >1000000 bp	178	178
Total length	3,225,940,482	3,222,774,051
Total length of scaffolds >1000000bp	3,171,591,131	3,168,695,343
Longest scaffold	145,919,952	145,820,377
N50	50,640,186	50,627,973
Mapping rate /%	99.76	99.77
Depth	19.16	19.18
Structural error	128	129
Expansion	92	37
Collapse	30	52
Haplotype switch	3	36
Inversion	3	4
Small-scale assembly error /per Mbp	18.34224789	2.43827208351
Total small-scale assembly error	59171	7858
Base substitution	38003	7394
Small-scale expansion	9509	232
Small-scale collapse	11659	232
QV	44.69560439	48.4526948745
**BUSCO**		
C (%)	91.50%	92%
Complete BUSCOs (C)	12.614	12,676
Complete and single-copy BUSCOs (S)	12.391	12,455
Complete and duplicated BUSCOs (D)	223	221

Fragmented BUSCOs (F)	185	155
Missing BUSCOs (M)	981	949
Total BUSCO groups searched	13,780	13,780

Table S8. Assembly quality comparison of VHG1.1 and VHG1.2

**Parameters**	**VHG1.1**	**VHG1.2**
**JASPER**		
QV	38.26542	42.89614
**QUAST**		
# contigs	295	295
GC (%)	40.85	40.85
# misassemblies	2.072	5.773
Total length	3,222,774,051	3,222,784,990
Unaligned length	21,898,542	21,246,362
Largest contig	14,582,0377	14,5820,867
N50	50,627,973	50,628,337
# N's per 100 kbp	89,219	90,796
# mismatches per 100 kbp	11,609.83	11,609.8

Table S9. Arrangement of super-scaffolds into chromosomal-level scaffolds

Chromosome (chr)	Super-Scaffold	Length (bp)	Total length (bp)
Chr1	Super-Scaffold_103810	358,187	277,207,185
	Super-Scaffold_2160	489,763	
	Super-Scaffold_102541	500,314	
	Super-Scaffold_104102	310,581	
	Super-Scaffold_29	10,207,602	
	Super-Scaffold_1115	11,400,731	
	Super-Scaffold_367	100,359,851	
	Super-Scaffold_2194	576,635	
	Super-Scaffold_1608	2,852,028	
	Super-Scaffold_55	5,608,690	
	Super-Scaffold_546	11,880,259	
	Super-Scaffold_100141	41,349,598	
	Super-Scaffold_100142	44,193,168	
	Super-Scaffold_100321	4,291,708	
	Super-Scaffold_100322	22,390,784	
	Super-Scaffold_1512	20,437,286	
Chr2	Super-Scaffold_100151	87,186,184	311,930,377
	Super-Scaffold_100152	71,507,842	
	Super-Scaffold_1860	2,431,530	
	Super-Scaffold_1817	1,369,078	
	Super-Scaffold_299	676,920	

	Super-Scaffold_486	145,919,830	
	Super-Scaffold_2017	1,076,410	
	Super-Scaffold_102321	194,433	
	Super-Scaffold_101751	1,182,589	
	Super-Scaffold_101752	265,552	
	Super-Scaffold_103391	120,009	
Chr3	Super-Scaffold_1449	90,761,156	223,710,811
	Super-Scaffold_100021	34,579,035	
	Super-Scaffold_100022	95,400,561	
	Super-Scaffold_5873	2,970,059	
Chr4	Super-Scaffold_1667	1,520,754	188,407,098
	Super-Scaffold_445	1,263,059	
	Super-Scaffold_2045	6,437,735	
	Super-Scaffold_100601	39,838,743	
	Super-Scaffold_103881	603,226	
	Super-Scaffold_3666	138,321,837	
	Super-Scaffold_1763	421,744	
Chr5	Super-Scaffold_394	46,959,862	178,820,811
	Super-Scaffold_1619	19,590,010	
	Super-Scaffold_1689	103,330,586	
	Super-Scaffold_100902	502,863	
	Super-Scaffold_100901	7,017,373	
	Super-Scaffold_106652	498,608	

	Super-Scaffold_102331	921,509	
Chr6	Super-Scaffold_1407	58,464,766	169,595,975
	Super-Scaffold_1132	111,131,209	
Chr7	Super-Scaffold_1650	583,363	186,375,001
	Super-Scaffold_2189	390,434	
	Super-Scaffold_102032	246,675	
	Super-Scaffold_102031	587,271	
	Super-Scaffold_100742	1,327,278	
	Super-Scaffold_100741	3,866,612	
	Super-Scaffold_1473	50,640,341	
	Super-Scaffold_2123	686,733	
	Super-Scaffold_1127	1,768,904	
	Super-Scaffold_1535	1,239,869	
	Super-Scaffold_2159	1,101,836	
	Super-Scaffold_101392	656,480	
	Super-Scaffold_101391	1,491,042	
	Super-Scaffold_3529	555,745	
	Super-Scaffold_100561	2,782,351	
	Super-Scaffold_100562	4,564,063	
	Super-Scaffold_102850	127,670	
	Super-Scaffold_366	28,958,167	
	Super-Scaffold_104990	240,686	
	Super-Scaffold_100262	28,002,532	

	Super-Scaffold_100261	28,797,079	
	Super-Scaffold_100591	18,462,940	
	Super-Scaffold_1506	5,297,699	
	Super-Scaffold_1239	2,731,843	
	Super-Scaffold_101771	1,267,388	
Chr8	Super-Scaffold_2016	2,263,957	144,149,689
	Super-Scaffold_1313	41,355,124	
	Super-Scaffold_1595	41,235,937	
	Super-Scaffold_1593	54,771,637	
	Super-Scaffold_1978	1,069,520	
	Super-Scaffold_1866	1,030,490	
	Super-Scaffold_1717	1,225,514	
	Super-Scaffold_917	527,813	
	Super-Scaffold_104291	261,303	
	Super-Scaffold_103891	408,394	
Chr9	Super-Scaffold_1243	39,580,957	123,318,295
	Super-Scaffold_103001	486,214	
	Super-Scaffold_3825	1,476,598	
	Super-Scaffold_103490	426,548	
	Super-Scaffold_1265	1,623,330	
	Super-Scaffold_102041	754,746	
	Super-Scaffold_103410	291,147	
	Super-Scaffold_5773	2,195,038	

	Super-Scaffold_118	61,678,016	
	Super-Scaffold_1714	3,962,221	
	Super-Scaffold_104791	122,380	
	Super-Scaffold_102840	387,872	
	Super-Scaffold_1630	1,936,467	
	Super-Scaffold_104550	155,964	
	Super-Scaffold_104430	167,598	
	Super-Scaffold_1330	928,828	
	Super-Scaffold_3575	741,169	
	Super-Scaffold_3730	3,154,195	
	Super-Scaffold_1377	2,073,480	
	Super-Scaffold_102732	483,555	
	Super-Scaffold_1100	691,972	
Chr10	Super-Scaffold_1553	38,810,617	170,691,952
	Super-Scaffold_3751	848,337	
	Super-Scaffold_101851	527,051	
	Super-Scaffold_102051	712,468	
	Super-Scaffold_102212	326,691	
	Super-Scaffold_1403	570,717	
	Super-Scaffold_101062	2,173,165	
	Super-Scaffold_1472	42,872,997	
	Super-Scaffold_106262	42,885,924	
	Super-Scaffold_100511	39,324,204	

	Super-Scaffold_2079	1,353,942	
	Super-Scaffold_103050	285,839	
Chr11	Super-Scaffold_1538	229,814	144,415,512
	Super-Scaffold_1780	616,772	
	Super-Scaffold_2001	761,023	
	Super-Scaffold_1267	49,164,497	
	Super-Scaffold_1771	623,590	
	Super-Scaffold_1873	6,875,122	
	Super-Scaffold_1682	10,178,619	
	Super-Scaffold_1211	23,470,498	
	Super-Scaffold_105221	39,160,553	
	Super-Scaffold_105222	13,335,024	
Chr12	Super-Scaffold_100202	32,670,757	163,272,453
	Super-Scaffold_100201	34,139,775	
	Super-Scaffold_1184	92,788,429	
	Super-Scaffold_1101	3,673,492	
Chr13	Super-Scaffold_1248	96,267,278	96,936,502
	Super-Scaffold_1982	531,367	
	Super-Scaffold_104670	137,857	
Chr14	Super-Scaffold_1167	89,672,005	96,384,643
	Super-Scaffold_1711	1,569,142	
	Super-Scaffold_1393	816,764	
	Super-Scaffold_182	1,376,558	

	Super-Scaffold_1270	1,896,491	
	Super-Scaffold_1772	1,053,683	
Chr15	Super-Scaffold_2102	468,966	138,689,298
	Super-Scaffold_3577	9,565,639	
	Super-Scaffold_100831	1,693,157	
	Super-Scaffold_100081	69,477,444	
	Super-Scaffold_100082	56,221,174	
	Super-Scaffold_100832	620,872	
	Super-Scaffold_101821	399,309	
	Super-Scaffold_104411	242,737	
Chr16	Super-Scaffold_103111	400,360	88,121,252
	Super-Scaffold_1092	9,624,650	
	Super-Scaffold_1431	11,429,683	
	Super-Scaffold_1583	12,839,105	
	Super-Scaffold_104331	396,257	
	Super-Scaffold_104332	158,436	
	Super-Scaffold_1426	4,777,987	
	Super-Scaffold_1343	364,217	
	Super-Scaffold_1642	997,261	
	Super-Scaffold_1544	1,361,771	
	Super-Scaffold_1222	39,582,904	
	Super-Scaffold_1142	3,146,474	
	Super-Scaffold_1880	1,094,591	

	Super-Scaffold_103201	614,228	
	Super-Scaffold_102372	376,989	
	Super-Scaffold_102751	147,538	
	Super-Scaffold_1194	352,649	
	Super-Scaffold_104300	176,067	
	Super-Scaffold_102980	280,085	
Chr17	Super-Scaffold_1484	696,736	83,075,240
	Super-Scaffold_102141	230,210	
	Super-Scaffold_1568	15,787,463	
	Super-Scaffold_3843	2,306,684	
	Super-Scaffold_1577	5,126,639	
	Super-Scaffold_1452	10,169,945	
	Super-Scaffold_1415	7,331,851	
	Super-Scaffold_100622	67,067	
	Super-Scaffold_382	35,782,711	
	Super-Scaffold_101141	1,869,911	
	Super-Scaffold_1518	3,120,805	
	Super-Scaffold_102901	585,218	
Chr18	Super-Scaffold_100411	14,416,402	78,449,595
	Super-Scaffold_100412	2,825,290	
	Super-Scaffold_393	1,567,478	
	Super-Scaffold_1430	59,640,425	
Chr19	Super-Scaffold_103352	508,650	58,073,371

	Super-Scaffold_104520	175,371	
	Super-Scaffold_1168	330,864	
	Super-Scaffold_1336	322,564	
	Super-Scaffold_104160	146,883	
	Super-Scaffold_103241	316,379	
	Super-Scaffold_213	5,733,330	
	Super-Scaffold_101111	768,825	
	Super-Scaffold_101451	1,544,086	
	Super-Scaffold_225	1,164,655	
	Super-Scaffold_101352	532,913	
	Super-Scaffold_101351	1,186,387	
	Super-Scaffold_1829	554,911	
	Super-Scaffold_100	894,493	
	Super-Scaffold_1730	3,023,579	
	Super-Scaffold_103700	84,579	
	Super-Scaffold_102920	517,723	
	Super-Scaffold_100652	2,852,931	
	Super-Scaffold_100651	5,667,336	
	Super-Scaffold_1353	1,937,902	
	Super-Scaffold_1671	6,841,766	
	Super-Scaffold_1824	1,140,359	
	Super-Scaffold_1188	10,592,457	
	Super-Scaffold_1123	5,676,645	

	Super-Scaffold_1282	1,933,025	
	Super-Scaffold_1166	511,339	
	Super-Scaffold_2130	2,860,589	
	Super-Scaffold_101382	82,855	
	Super-Scaffold_104221	169,975	
Chr20	Super-Scaffold_1466	26,423,504	63,598,429
	Super-Scaffold_1531	812,167	
	Super-Scaffold_1310	1,893,617	
	Super-Scaffold_2049	350,712	
	Super-Scaffold_1264	2,171,700	
	Super-Scaffold_1478	29,626,810	
	Super-Scaffold_1840	1,313,058	
	Super-Scaffold_102691	342,454	
	Super-Scaffold_1894	664,407	
Chr21	Super-Scaffold_93	364,063	45,314,271
	Super-Scaffold_1985	1,042,444	
	Super-Scaffold_1532	1,880,208	
	Super-Scaffold_1757	1,457,671	
	Super-Scaffold_379	1,499,833	
	Super-Scaffold_1902	371,680	
	Super-Scaffold_117	30,422,572	
	Super-Scaffold_549	3,191,424	
	Super-Scaffold_102302	864,784	

	Super-Scaffold_102301	331,361	
	Super-Scaffold_1327	1,027,315	
	Super-Scaffold_102931	334,380	
	Super-Scaffold_102932	453,056	
	Super-Scaffold_1703	2,073,480	
Chr22	Super-Scaffold_4389	1,123,980	39,333,859
	Super-Scaffold_2191	1,884,919	
	Super-Scaffold_1180	1,091,997	
	Super-Scaffold_101921	980,985	
	Super-Scaffold_101922	400,853	
	Super-Scaffold_100462	2,808,247	
	Super-Scaffold_100461	15,715,072	
	Super-Scaffold_1715	6,099,510	
	Super-Scaffold_1240	3,706,663	
	Super-Scaffold_1225	1,412,669	
	Super-Scaffold_2034	1,239,371	
	Super-Scaffold_101991	1,123,784	
	Super-Scaffold_104730	145,821	
	Super-Scaffold_102561	488,686	
	Super-Scaffold__468	321,880	
	Super-Scaffold__432	789,422	
ChrX	Super-Scaffold_1114	3,768,266	139,221,676
	Super-Scaffold_1566	3,871,327	

	Super-Scaffold_100911	2,437,950	
	Super-Scaffold_1422	2,331,495	
	Super-Scaffold_1443	3,829,617	
	Super-Scaffold_2039	3,571,250	
	Super-Scaffold_1170	24,853,254	
	Super-Scaffold_105391	1,029,117	
	Super-Scaffold_105681	284,135	
	Super-Scaffold_1935	4,164,441	
	Super-Scaffold_1521	3,295,738	
	Super-Scaffold_1210	4,892,356	
	Super-Scaffold_101731	1,130,360	
	Super-Scaffold_1263	3,781,657	
	Super-Scaffold_1416	9,425,444	
	Super-Scaffold_1459	18,227,570	
	Super-Scaffold_1409	13,347,203	
	Super-Scaffold_1631	827,343	
	Super-Scaffold_1420	2,026,238	
	Super-Scaffold_1153	8,801,070	
	Super-Scaffold_1609	5,480,148	
	Super-Scaffold_1586	10,654,925	
	Super-Scaffold_1643	2,926,555	
	Super-Scaffold_1726	1,383,094	
	Super-Scaffold_1143	729,125	

	Super-Scaffold_729	441,137	
	Super-Scaffold_101431	1,710,861	
ChrY	Super-Scaffold_1278	1,565,907	9,744,533
	Super-Scaffold_102171	340,062	
	Super-Scaffold_1549	4,192,305	
	Super-Scaffold_103031	347,867	
	Super-Scaffold_2072	788,386	
	Super-Scaffold_101932	1,067,913	
	Super-Scaffold_1325	1,442,093	
Undetermined	Super-Scaffold_1260	216,582	8,781,447
Undetermined	Super-Scaffold_370	418,002	
Undetermined	Super-Scaffold_1530	787,065	
Undetermined	Super-Scaffold_218	369,491	
Undetermined	Super-Scaffold_2105	795,234	
Undetermined	Super-Scaffold_104392	1,049,176	
Undetermined	Super-Scaffold_104172	441,348	
Undetermined	Super-Scaffold_1159	1,106,364	
Undetermined	Super-Scaffold_1976	1,077,070	
Undetermined	Super-Scaffold_1705	1,185,846	
Undetermined	Super-Scaffold_2138	527,538	
Undetermined	Super-Scaffold_2015	807,731	
Total length		3,227,619,275	

Table S10. Summary of the VHG1.2 genome assemblies using Minimap2

Identity	% aligned	
	**hg38**	**T2T**
no match	13.47	6.17
<25%	0.09	0.34
<50%	0.16	0.89
<75%	07.03	6.18
>75%	79.25	85.88

Table S11. Comparison of the number of SNPs and indels in three Vietnamese genomes from the 1000 Genomes Project, using VHG and hg38 as reference genomes

Kinh genome	Reference	GATK4.4.0.0		Deepvariant	
		No. of SNPs	No. of indels	No. of SNPs	No. of indels
HG02079	VGH1.2	2,399,319	377,878	3,371,678	582,66
	VGH1.1	2,527,145	389,559	3,491,742	597,79
	hg38	2,765,419	459,148	3,666,387	65,423
HG02070	VHG1.2	3,125,906	553,931	3,219,650	632,58
	VHG1.1	3,269,881	567,799	3,345,714	647,2
	hg38	3,283,403	594,443	3,501,306	693,45
HG01852	VHG1.2	2,332,002	348,006	2,633,143	385,271
	VHG1.1	2,457,713	359,35	2,751,199	397,758
	hg38	2,649,475	409,417	2,945,807	441,687

Table S12. P-values from pairwise comparisons of the number of detected variants between different reference genomes

	**P value**		
	**VHG1.2-VHG1.1**	**VHG1.1-hg38**	**VHG1.2-hg38**
No. of SNPs called by GATK	0.0019	0.1635	0.047
No. of indels called by GATK	0.0039	0.0592	0.0352
No. of SNPs called by DeepVariant	0.0004	0.0041	0.0009
No. of indels called by DeepVariant	0.0033	0.0061	0.0051
Note: Values of $p < 0.05$ were accepted as significant			

Table S13. Structural variant (SV) counts in three Vietnamese genomes from 1KGP, detected with Cue using VHG1.2 and GRCh38 as references

**Total**	**No. of SVs**		
	**HG02079**	**HG02070**	**HG01852**
No. of SV based on VHG1.2	26	17	18
No. of SV based on hg38	84	68	64
No. of SV based on vhg1.2 shared with other samples	7	4	4
No. of SV based on hg38 shared with other samples	34	31	29
% SV based on hg38 shared with other samples	40.5	45.6	45.3
% SV based on vhg1.2 shared with other samples	26.9	23.5	22.2
% total shared SV	37.6	41.7	40.7
% shared between all three total	13.6	17.6	18.3
% SV based on hg38 shared between all three sample	11.5		
% SV based on vhg1.2 shared between all three sample	2.2		

Table S14. Alignment summary of VHG1.2 with other assemblies (Han Chinese (HX1), Korean (AK1), and Japanese (JG1)) using Minimap2

**Identity**	**% alignment**			
	**AK1**	**HX1**	**JG1**	**hg38**
no match	5.49	6.62	10.68	13.47
<25%	0.02	0.05	0.03	0.09
<50%	0.2	0.32	0.2	0.16
<75%	7.35	8.43	6.79	7.03
>75%	86.95	84.58	82.29	79.25
